# Future of Bariatric Embolization: A Review of Up-to-date Clinical Trials

**DOI:** 10.7759/cureus.7958

**Published:** 2020-05-04

**Authors:** Israa Aldawudi, Prakash C Katwal, Srood Jirjees, Zin Mar Htun, Safeera Khan

**Affiliations:** 1 Radiology, California Institute of Behavioral Neurosciences and Psychology, Fairfield, USA; 2 Internal Medicine, California Institute of Behavioral Neurosciences and Psychology, Fairfield, USA; 3 Neurology, California Institute of Behavioral Neurosciences and Psychology, Fairfield, USA; 4 Internal Medicine, California Institute of Behavioral Neuroscience and Psychology, Fairfield, USA

**Keywords:** bariatric embolization, left gastric artery embolization, ghrelin hormone, bariatric surgery/therapeutic use"[mesh], obesity management, interventional radiology, bariatric surgery, metabolic syndrome, diabetes, obesity

## Abstract

Obesity is a significant health issue with an overall rise in mortality; it has multiple risk factors, including hormonal effects, which play a significant role in the balance of food intake and weight gain. Ghrelin is an anabolic hormone secreted from stomach fundus and plays a significant role in this regulation. Management of obesity involves multiple interventions, including lifestyle adjustment, pharmacotherapy, and bariatric surgery. Bariatric embolization is a relatively new procedure; several animal studies show that embolization of the left gastric artery reduces serum ghrelin and induces weight loss.

Also, several clinical studies were conducted in the past ten years which have shown bariatric embolization's effectiveness in inducing weight loss: a meta-analysis of 47 patients included in six different clinical studies of left gastric artery embolization resulted in 8% total weight loss from baseline body weight. Many studies also show this procedure's effect on lowering the HgA1C level and lipid profile. Clinical studies mostly reported minor adverse effects such as transient abdominal discomfort, nausea and vomiting, gastric ulcers, and major adverse effects were uncommon, suggesting the procedure is well tolerated. It may be an alternative line of management in patients who are not suitable candidates for bariatric surgery.

Although future clinical studies will provide an answer to several questions like the exact effects of the procedure on diabetes and metabolic syndrome, future studies are also needed to establish particular guidelines to match different patient characteristics with their optimal procedural techniques and pre- and post-procedure evaluation tests.

## Introduction and background

Adult obesity is defined as a body mass index (BMI) of 30 kg/m^2^ or higher, while BMI of 25 to 29 kg/m^2^ is defined as overweight [1]. Obesity is a major health issue worldwide; the prevalence of obesity in adults rising to 27.5% [2], with an overall mortality increase by 29% [3], for every five-unit increase in BMI above 25 kg/m2 [4].

Obesity-related comorbid conditions on multiple body systems can induce premature mortality and cardiovascular disease; metabolic diseases like Type 2 diabetes, dyslipidemia, and nonalcoholic fatty liver disease; respiratory diseases like obstructive sleep apnea and asthma; reproductive and genitourinary conditions like infertility, sexual dysfunction, stress urinary incontinence; moreover, generally impaired quality of life and increased risk of cancer[5]. Obesity has multifactorial risk factors, including numerous polymorphic gene products, gut microbiome, chronodisruption, and hormone effects [6]. However, the exact cause of obesity is not clear [7], Hormonal effects play a major role in the balance of food intake and weight gain by sending signals to multiple areas in CNS responsible for appetite regulation [8-9].

The hormones involved in this regulation are glucagon-like peptide, oxyntomodulin, peptide tyrosine-tyrosine, cholecystokinin, adiponectin [8-9], as well as ghrelin, which is the only gastrointestinal anabolic hormone that produces positive energy and increases body weight gain [10-11]. Ghrelin is an acylated peptide hormone that exists in two forms, namely acylated ghrelin (AG) and unacylated ghrelin O-acyltransferase (GOAT). The latter transfers an acyl group from the fatty acids to the serine-3 residue of ghrelin, which works to stimulate the appetite [12-13]. Therefore, if GOAT activity is blocked, it will induce weight loss [12, 14]. Ghrelin is mainly secreted by secretory granules of submucosal cells of the fundus of the stomach [15], and its levels can be influenced by multiple factors including diet, lifestyle, exercises, and environment [16].

Management of obesity involves various lifestyle interventions, pharmacotherapy, and bariatric surgery. Also, several animal studies have shown that embolization of the left gastric artery will decrease serum ghrelin hormone level secretion and evenly reduce body weight [17-19].

The failure of medical intervention, obesity-related comorbidities, as well as specific BMI criteria that reflect the severity of obesity are the indications for bariatric surgery [20-21]. Weight loss is the main objective of bariatric surgery and has been uniformly reported [22], although this procedure is associated with preoperative surgical complications, perioperative surgical mortality, and long-term complications [5]. On the other hand, gastric embolization is a relatively new procedure and has shown positive results, but there is not much data about the comparison of both techniques.

Will bariatric embolization be considered an alternative to bariatric surgery in the treatment of obese patients in the near future? In an attempt to answer this question, this review article will analyze and compare the efficacy and safety of both procedures by reviewing the clinical trials involving bariatric artery embolization from the past ten years.

## Review

Methods

We used the PubMed and Medline database to collect data using the following keywords: bariatric embolization, left gastric artery embolization, ghrelin hormone, and Bariatric Surgery/therapeutic use"[Mesh].

Inclusion/Exclusion Criteria

We thoroughly searched for all types of articles relevant to the topic and only included papers published in English between 2014 to 2020. Also, only full-text articles were selected. All articles were finalized after going through the full text. Animal studies were not included and no geographical location was specified while choosing articles. Quality checks were not performed for all articles. Among articles included, we had nine clinical trials, one systematic review, and a meta-analysis.

Discussion

Trans-arterial left gastric artery embolization via embolic microspheres leads to local ischemia of the stomach's fundus and suppresses the anabolic hormone function, which then decreases appetite and reduces weight. Several clinical trials were conducted in the past ten years that showed promising short term results [23].

To discuss the efficiency of bariatric embolization in reducing weight and to assess the safety of the procedure, we included clinical studies published from 2014 to 2020. Six prospective clinical studies were about obesity, and three retrospective studies discussing upper GI bleeding were included. More details about the studies are shown in Table 1. 

**Table 1:** Baseline patients' characteristics from the included studies N/A= Not available

First author, year	Simple size	Age	Female %	Baseline BMI	Baseline weight kg	Access | catheter
Obesity Clinical Trials						
Weiss et al., 2019 [23]	15	44 ± 11	80%	45 ± 4.1	139 ± 20	Femoral artery 5 F SOS
Elens et al. 2019 [24]	11	38 ± 10	87%	28.9+ 2.5	79 ± 10	Femoral artery 5 F Cobra
Pirlet et al. 2019 [25]	7	48 ± 7	0	52 ± 8	160 ± 27	Radial artery 5 Fr 125 cm JR 5
Bai et al., 2018 [26]	5	42.8 ± 13.9	40%	38.12 ± 3.8	102 ± 16	Femoral artery 5 Fr
Syed et al., 2016 [27]	4	41.0 ± 9.2	70%	42.4 ± N/A	118 ± N/A	Femoral | Radial 4|5 Fr Simmons 1
Kipshidze et al., 2015 [28]	5	44.7 ± 7.4	20%	42.2 ± 6.8	128 ± 24	Femoral artery 6 Fr JR 4
Upper GI Bleeding Studies						
Takahashi et al., 2019 [29]	16	57.6 ± 12.9	44%	30.0 ± 4.3	87.9 ± 12.5	N/A
Kim et al., 2018 [30]	21	56.3 ± 15.6	43%	29.9 ± N/A	93 ± N/A	Femoral artery 5 F SOS
Gunn, et al. 2014 [31]	19	64.6 (46–92)	37%	30.0	N/A	N/A

The Efficiency of Left Gastric Artery Embolization 

A meta-analysis by Hafezi et al., involving 47 patients from six prospective clinical trials showed that a mean weight loss in kilogram (kg), observed after an average follow-up time period of 12 months following left gastric embolization (LGE) procedure, was 8.68 kg +/- 1.24 kg (p< .001), approximately 8% of baseline total body weight [32]. Comparing it to other procedures, the percentage of weight loss was up to 36% after roux-en-y gastric bypass [33], and 19% by gastric banding [34], whereas, weight loss due to diet and exercise, is less than 5% of baseline body weight [35-36].

Retrospective studies conducted by Takahashi et al., Kim et al., and Gunn et al. [29-31], that discussed LGE for management of upper GI bleeding also concluded that patients who underwent an LGE procedure for the management of Upper GI bleeding, had a significant weight loss. All studies concluded the same results; showing bariatric embolization is effective in inducing weight loss.

Mean weight loss percentage and mean weight loss in kilograms for each study are mentioned in Table 2.

**Table 2:** Results from the including studies showing weight loss after left gastric embolization procedure M= Months N/A= Not available

Author, year	Follow-up period (in months)	Mean weight loss in kg	Total weight loss Percent
Weiss et al. 2019 [23]	15	7.62	5.96 %
Elens et al., 2019 [24]	6	8.0	10.0 %
Pirlet et al., 2019 [25]	12	13.0	4.77 %
Bai et al., 2018 [26]	9	12.90	12.64 %
Syed et al, 2016 [27]	6	9.19	8.52 %
Kipshidaze et al, 2015 [28]	20-24	22.0	17.19 %
Takahashi et al., 2019 [29]	1.5	6.4	6.3 %
Kim et al., 2018 [30]	12	16.3	N/A
Gunn et al., 2014 [31]	13.6	N/A	N/A

A study by Pirlet et al. was the only study included that had 100% of morbidly obese male participants whereas, in the study by Kipshidze et al., males made up 80% of the participants; these two studies have a slightly higher percentage of weight loss compared to the other prospective studies included [25, 28]. A study by Elen et al. was the only clinical trial studied the LGE in overweight participants with a mean baseline BMI of 27.4 kg, resulting in 10% weight loss of their total body mass [32]. These findings may suggest that male gender and lower baseline BMI are favorable factors regarding the efficiency of the procedure. 

Regarding the efficiency of the LGE procedure on the reduction of the ghrelin hormone levels, several findings from published clinical trials that were relevant to our research question were extracted for the review. The maximum ghrelin reduction percentage in the study by Bai et al. [26], is 41+/- 13%, and 36+/- 4% in the study by Kipshidze et al. [28], while in Syed et al., two of four participants had a decrease in their ghrelin baseline level after six months of follow up with the total ghrelin percentage -5+/- 22% [27]. Whereas, in comparison, up to 40-50% reduction of total serum ghrelin was observed after sleeve gastrectomy after a mean follow up period of five years [37]. 

The effect of LGE procedure on lowering lipid profile, HgA1C, and metabolic syndrome is not clear, although some clinical trials show promising results. A clinical trial by Weiss et al. resulted in significant metabolic changes after 12 months of follow up in levels of total cholesterol, low-density lipoprotein, mean blood glucose, and HgA1C, independent of weight loss. Also, a clinical trial conducted by Syed et al [27], showed similar results of lower HgA1C levels at third and sixth months of follow-up after the procedure in obese diabetic participants, which suggests that LGE procedure has some effect on metabolism. 

More studies are needed to determine the exact effect of LGE on lipid profile, diabetes, and metabolic syndrome. Future studies may also provide an answer to several questions like does the combination of LGE procedure with other modalities of weight regulation, will increase their efficiency and effects, either on weight loss or on metabolic syndrome. Also, further studies would possibly shed light on certain instructions or guidelines to follow, both before and after the procedure, to achieve the maximum impact of the procedure on weight loss.

Safety of Left Gastric Artery Embolization 

Bariatric embolization preliminary clinical trials included in this review reported minor adverse effects after the procedure; these clinical trials are summarized in Table 3. In a study by Elens et al., only one patient out of 16 participants developed a major complication, i.e. pancreatitis associated with splenic infarction and gastric perforation [24]. On the other hand, one out of 21 patients in the clinical trial by Kim et al., reported death related to the medical history of the patient [30]. 

**Table 3:** Studies reviewed to assess LGE safety PPI: proton pump inhibitor N/A: not available

Author, Year	Follow-up, evaluation, and preventative measures	Complications	Treatment
Weiss et al., 2019 [23]	Upper endoscopy before and three days after the procedure. Upper endoscopy after 30 days of the procedure for participants who had a positive finding.	Mild nausea, transient vomiting and mild epigastric pain in three patients immediately after the procedure as well as superficial gastric ulcer in three patients on the third day after the procedure.	PPI one week before the procedure and one week after.
Elens et al., 2019 [24]	Upper endoscopy one to two months after the procedure in 10 participants.	One patient developed pancreatitis associated with splenic infarction and gastric perforation, and another patient developed a superficial gastric ulcer.	One month of hospital stay and ICU admission; pantoprazole (40 mg) a day for six weeks.
Pirlet et al., 2019 [25]	PPI for 24 hours	Transient abdominal discomfort in six patients.	N/A
Bai et al., 2018 [26]	Upper endoscopy on the day of the procedure, three and 30 days after for participants who had positive findings. Prophylactic Omeprazole for two weeks after the procedure.	Four participants developed mild epigastric discomfort. One patient had a superficial gastric ulcer and another patient had punctured hematoma which is resolved after 22 days.	N/A
Syed et al. 2016 [27]	Upper endoscopy before the procedure, and third day after. Upper endoscopy after 30 days of the procedure for participants with positive findings. PPI one week before to one month after the procedure.	Nausea and vomiting, mild epigastric pain immediately after the procedure, and only three participants developed a gastric ulcer.	N/A
Kipshidze et al., 2015 [28]	Two upper endoscopies are performed on participants' first day as well as one week after the procedure.	Three participants had mild abdominal discomfort after the procedure.	N/A
Takahashi et al., 2019 [29]	N/A	N/A	N/A
Kim et al., 2018 [30]	Six participants underwent upper endoscopy after the procedure.	Gastric ulcer in two patients and jejunal ischemia in one patient due to the holding of the anticoagulant. Death in one patient within two months of the procedure due to heart failure medical complications.	N/A
Gunn et al., 2014 [31]	N/A	N/A	N/A

Preliminary clinical studies show that LGE is a safe and well-tolerated procedure [23-28], but the safety and effectiveness of the LGE procedure, if performed for a second time after a certain time period, is not established.

Embolization techniques, including arterial access, catheter types, shape, and size of microparticles, are different in clinical studies mentioned in Table 1. The pre- and post-procedure evaluation and prevention tests also vary in each of the studies, summarized in Table 3. These technical and procedural evaluations and follow-up test variations suggest that certain guidelines are needed to establish the maximum effectiveness and safety of each LGE procedure based on each participant’s overall criteria and risk factors. 

Study Limitations 

The duration of included clinical trials ranges between two months to 48 months, whereas, to evaluate the efficiency and safety of the procedure at least five years of follow up is needed. The baseline BMI and other participant characteristics are different in each clinical study included in this review. Research results from animal studies and pathophysiology of procedures are not discussed in this paper. More clinical trials in the future with longer follow-up periods will give better insights into the efficiency of this procedure.

## Conclusions

Primary endpoint: The outcome of a preliminary clinical trial of left gastric artery embolization procedure demonstrates that the procedure is well tolerated and is effective in inducing weight loss; however, more studies are needed in the future to establish favorable baseline characteristics of the participants who will benefit the most from the procedure. The clinical studies included reported only minor adverse effects, and major complications were atypical.

Secondary endpoint: the preliminary studies of bariatric embolization propound that LGE will be an alternative line of management in patients who are not a candidate for bariatric surgery.

Tertiary endpoint: Furthermore, clinical studies are needed to establish particular guidelines to label the different participant's characteristics with their optimal procedural techniques and pre-and post-procedure evaluation tests. 
